# Microbiota and nutrition as risk and resiliency factors following prenatal alcohol exposure

**DOI:** 10.3389/fnins.2023.1182635

**Published:** 2023-06-15

**Authors:** Deepa Upreti, Siara K. Rouzer, Abigail Bowring, Emma Labbe, Rosaline Kumar, Rajesh C. Miranda, Amanda H. Mahnke

**Affiliations:** Department of Neuroscience and Experimental Therapeutics, Texas A&M University School of Medicine, Bryan, TX, United States

**Keywords:** nutrition, gut-brain axis, gut microbiota, ethanol, FASD, non-protein-coding RNA, extracellular vesicles

## Abstract

Alcohol exposure in adulthood can result in inflammation, malnutrition, and altered gastroenteric microbiota, which may disrupt efficient nutrient extraction. Clinical and preclinical studies have documented convincingly that prenatal alcohol exposure (PAE) also results in persistent inflammation and nutrition deficiencies, though research on the impact of PAE on the enteric microbiota is in its infancy. Importantly, other neurodevelopmental disorders, including autism spectrum and attention deficit/hyperactivity disorders, have been linked to gut microbiota dysbiosis. The combined evidence from alcohol exposure in adulthood and from other neurodevelopmental disorders supports the hypothesis that gut microbiota dysbiosis is likely an etiological feature that contributes to negative developmental, including neurodevelopmental, consequences of PAE and results in fetal alcohol spectrum disorders. Here, we highlight published data that support a role for gut microbiota in healthy development and explore the implication of these studies for the role of altered microbiota in the lifelong health consequences of PAE.

## 1. Introduction

Alcohol use is common among adults of reproductive age, and moreover, as with other psychotropic drugs, alcohol can be addictive and abstention aversive. In 2020 it was estimated that 60.7% of young adults (ages 18–25) and 55.1% of adults (26 years of age or older) consumed alcohol within the last month, with 44.4% of all alcohol consumers engaging in binge drinking (Substance Abuse and Mental Health Services Administration, 2021). Alongside these high rates of alcohol use in individuals of child-bearing age, the United States also has high rates of unintended pregnancy. Data from 2008 to 2011 show that for individuals between the ages of 18–29 years, i.e., the peak reproductive years, between 66 and 81% of all pregnancies are unintended (Finer and Zolna, 2016). More recent estimates, from the Center for Disease Control’s Pregnancy Risk Assessment Monitoring System 2020 data, indicated that 28.5% of pregnancies resulting in live births were unintended (America's Health Rankings, 2023), which is comparable to the 22% of live births occurring from unintended pregnancies from the 2008–2011 dataset (Finer and Zolna, 2016). Rates of unintended pregnancy can vary widely across states, ranging from 19.4 to 41.0% of live births (America's Health Rankings, 2023). These data collectively suggest that pregnancy awareness varies, particularly during early pregnancy and peak reproductive years, when alcohol use occurs at the highest rates, resulting in the common occurrence of prenatal alcohol exposure (PAE). Several North American state-wide assessments of alcohol metabolites in neonatal blood (Bakhireva et al., 2017; Umer et al., 2020; DiBattista et al., 2022), indicate that between 8 and 15% of infants in the general population are exposed to alcohol in the last month of pregnancy. Binge alcohol exposures, the pattern of drinking for almost half of alcohol consumers, can be particularly harmful for *in utero* fetal development (Bonthius and West, 1990), although cessation of this drinking pattern can be moderately protective against negative neurobehavioral outcomes compared to consumption throughout pregnancy, even at low, albeit sustained, levels (Bandoli et al., 2022). Therefore, it is not surprising that fetal alcohol spectrum disorders (FASDs), the collection of neurodevelopmental and growth deficits that can occur following PAE (Popova et al., 2023), are prevalent, occurring in a conservatively estimated 1.1–5% (weighted estimate of 3.1–9.9%) of school-aged children in the United States (May et al., 2018). The implications are that PAE constitutes a health risk factor for up to 1 in 10 of all US children and adults.

**Figure 1:**
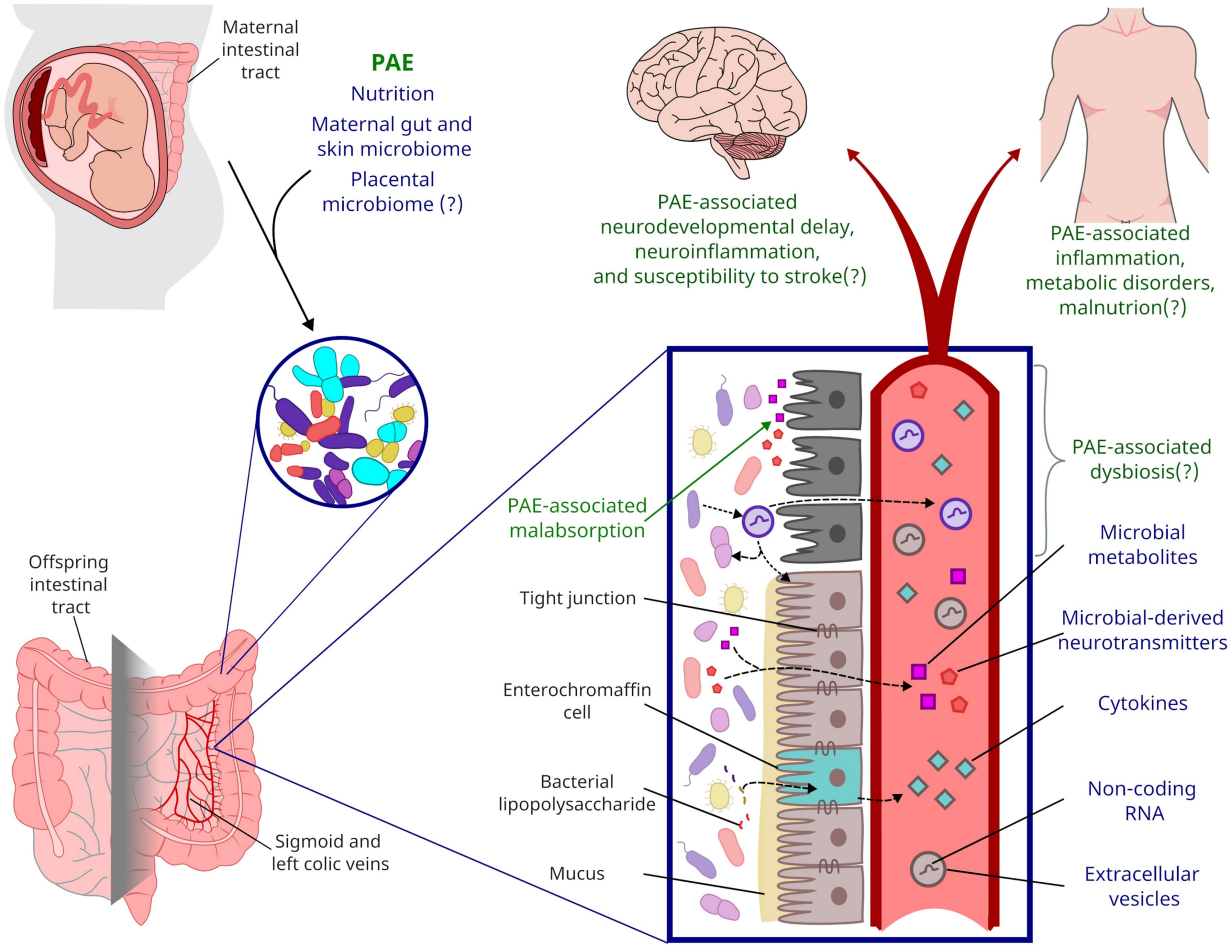
Framework for the life-long impact of PAE on gut microbiota. During parturition, a neonate’s gastrointestinal microbiota is colonized from maternal sources. PAE likely shapes the neonatal microbiome through alcohol’s impacts on maternal microbiota and nutrient absorption. PAE has been shown to impact microbial metabolites present in offspring circulation, but PAE may also impact inflammation and other endocrine factors through altered microbiota and gut function. Affectors and effectors of changes to microbiota discussed in this review are indicated by blue text while the potential life-long consequences of PAE are indicated by green text.

While the neurodevelopmental impacts of PAE have historically been well studied, there are also a large number of co-morbid conditions that can also occur, as has been shown by a systematic review with meta-analysis (Popova et al., 2016) and by a self-report survey of adults with FASDs (Himmelreich et al., 2020). Not all individuals with FASDs will develop these co-morbidities nor will all individuals with PAE develop a FASD. Preclinical research has shown that multiple factors, including nutrition, genetics, stress, and the timing of alcohol exposure during pregnancy (Petrelli et al., 2018), can confer risk or resiliency to the negative consequence of PAE. Critically, for other neurodevelopmental disorders, microbiota have been shown to not only be at the intersection of a number of these risk/resiliency factors, but also directly contribute to neurodevelopment through the gut-brain axis and, potentially, through the placenta-brain axis. Here, we provide a framework for the potential role of microbiota as a risk/resiliency factor following PAE, by integrating the literature on the known impacts of PAE on nutritional insufficiencies and microbiota with the more robust findings for the role of microbiota in other neurodevelopmental disorders. We also extrapolate what alterations to microbiota could mean for plasma biomarkers/mediators of PAE’s effects, long-term health for individuals with FASDs, and the therapeutic potential of targeting PAE-induced alterations to microbiota (Figure 1).

## 2. Microbiota and the developing brain

### 2.1. Gut microbiota

The microbiome is the genomic content of the community of microbes, known as the microbiota, that colonize human body surfaces, such as the skin, respiratory tract, genitourinary tract, and gastrointestinal tract. The gastrointestinal tract is extensively colonized with microbiota, with human gut flora estimated to contain greater than 10^13^ microbial cells, resulting in approximately a 1:1 microbial: human cell ratio (reviewed in Sender et al., 2016). Gut microbiota are predominantly bacteria, but also include archaea, viruses, and unicellular eukaryotes (reviewed in Sender et al., 2016; Thursby and Juge, 2017). Bacteria belonging to the Firmicutes and Bacteroidetes phyla make up the majority of the gut bacteria, whereas other phyla that are present in minor proportions include, but are not limited to, Fusobacteria, Verrucomicrobia, Proteobacteria, and Actinobacteria (reviewed in Sekirov et al., 2010).

The human gut microbiota is shaped by early life events such as the delivery method (vaginal delivery vs. cesarean section), maternal use of antibiotics, and feeding modality, e.g., breast-, formula-, or mixed-feeding (Niu et al., 2020). In a study that examined fecal microbial content from individuals across the lifespan (0–70 years of age) in three geographically, culturally, and nutritionally distinct communities, individuals ≤3 years of age had the greatest interindividual microbial variability (Yatsunenko et al., 2012). The adults in this study had less interindividual and intercommunity variability than the ≤3 years of age group. The intercommunity variability identified in this study was attributed to differences in nutrition, as the microbial content for the community with a predominantly carnivorous diet could be statistically segregated from the other two communities with predominantly herbivorous diets. A recent study has shown that for adults, microbial content can be stable over time (Frost et al., 2021). When microbial content was assessed from matched fecal samples, acquired at a median interval of 5 years, the three most abundant genera, *Bacteroides*, *Prevotella,* and *Faecalibacterium*, were maintained across this ~5 year time span. Variability in microbiota was almost entirely attributable to interpersonal differences, with intraindividual variability across the time span associated with the development of metabolic disorders. For adults, dietary changes can be a potent modifier of gut microbiota. For healthy adults administered a controlled high-fat/low-fiber or low-fat/high-fiber diet, although interindividual differences were the largest contributor to microbiota variability, all subjects showed a change in microbial content within the first 24 h (Wu et al., 2011). Similarly, 5 days of an animal product-based diet, labeled with a food-tracking dye, increased microbiome diversity within 24 h of the diet reaching the distal gut compared to baseline microbiome diversity (David et al., 2014). This effect was not seen with a plant-based diet. Additionally, the increase in microbiome diversity from the animal product-based diet was normalized 2 days after returning to their normal diet. Therefore, gut microbiota can stabilize across the lifespan but are sensitive to alterations due to dietary and physiological changes, suggesting that gut microbiota can be targeted for rapid intervention.

### 2.2. Gut-brain axis

The gut-brain axis is a complex bidirectional communication network between gut microbiota and the central nervous system. For example, through the autonomic nervous system signaling, the central nervous system can affect gut motility and secretion of bile and mucous, which can alter microbial colonization in the gut. Reciprocally, gut microbiota can shape human health through the production and release of metabolites into human circulation, impacting metabolic, immune, and neuroendocrine networks (Rhee et al., 2009).

Metabolic products released by gut microbiota include short-chain fatty acids (SCFAs), indole derivatives, choline metabolites, polyamines, and lipopolysaccharides (reviewed in Anand and Mande, 2018). SCFAs, such as acetate, propionate, and butyrate, are the major metabolic products of gut microbial anerobic fermentation. They can enter gut endothelial cells or actively be transported across the basement membrane to blood capillaries and into circulation (reviewed in van der Hee and Wells, 2021). A number of bacteria can also produce a range of mammalian neurotransmitters (reviewed in Strandwitz, 2018), release tryptophan and other indole derivatives that promote synthesis of neurotransmitters and neuropeptides by the endocrine enterochromaffin cells (reviewed in Gao et al., 2020). Microbiota also release byproducts, such as polyamines and lipopolysaccharides, that impact immune function both within the gut and throughout the body through the release of cytokines both by enterochromaffin cells and resident intestinal immune cells (Zheng et al., 2020).

The products released by microbiota can have systemic effects, including in the central nervous system, through multiple pathways. Microbiota-released SCFAs, as well as neurotransmitters and peptides synthesized by the endocrine enterochromaffin cells, can signal through G-protein coupled receptors on a variety of cells, including vagal nerve afferents (reviewed in Husted et al., 2017; van der Hee and Wells, 2021). This signaling through the vagal nerve can directly impact brain chemistry and neurobehavioral outcomes (Bravo et al., 2011; Goswami et al., 2018). Additionally, along with cytokines made by the local immune and endocrine cell populations, SCFAs can act as endocrine factors, signaling systemically in the host (Cryan and Dinan, 2012). Materials released by microbiota can cross the blood brain barrier, including extracellular vesicles (see 5.2, Cuesta et al., 2021) and some metabolites (reviewed by Cryan and Dinan, 2012; e.g. Needham et al., 2022). Gut microbiota can also alter blood brain barrier integrity, thereby making the brain more (or less) susceptible to microbial metabolites. In a germ-free mouse model, which has normally-occurring blood brain barrier permeability, colonization with SCFA-producing bacteria improved blood brain barrier integrity (Braniste et al., 2014). Therefore, not only can secreted factors from microbiota act peripherally to impact brain function, but some can also cross the blood brain barrier to act directly on the CNS.

Gut microbiota metabolites can also impact host gene expression through epigenetic mechanisms. Butyrate can inhibit histone deacetylase, thereby promoting histone acetylation and subsequent chromatin relaxation and gene expression (reviewed in Stilling et al., 2016). A recent clinical study found that reduced amounts of fecal butyrate, and butyrate-producing bacteria, were associated with increased depression symptoms in individuals with Parkinson’s disease (Xie et al., 2022). This study also identified leukocyte genes whose methylation correlated with fecal butyrate levels in patients. To connect these butyrate-associated changes in peripheral methylation to changes within the brain, the researchers compared the identified leukocyte genes to previous work that identified genes with altered methylation in prefrontal cortical neurons (Li et al., 2020). They found significant overlap and correlated likelihood of methylation in Parkinson’s disease patients for the leukocyte and neuronal genes, suggesting the potential for decreased butyrate levels to contribute to neurological changes through effects on brain DNA methylation (Xie et al., 2022). Choline is a dietary source of one-carbon units for DNA methylation. Gut microbiota can decrease choline bioavailability by metabolizing choline. When germ-free mice were colonized with *Escherichia coli* that use choline as a nutrition source, serum choline levels and DNA methylation were decreased, even with high choline supplementation in the diet (Romano et al., 2017). These choline-deficit mice also exhibited elevated markers of metabolic disorders, e.g., serum and live triglycerides (for choline deficits following PAE, see 4.1.2.1). Additionally, as discussed below (see 5.3.2), microbiota-derived extracellular vesicles can carry small RNA molecules that can potentially act like long-non-coding RNAs in recipient cells to impact gene expression.

### 2.3. Placenta-brain axis

Appropriate placental development and function is critical for fetal development and, ultimately, for the offspring’s long-term health. The placenta exchanges nutrients, gasses, and waste between the mother and the fetus, in addition to producing the hormones needed to support the fetus and maintain pregnancy (Godfrey, 2002). The placenta can also directly contribute to neurodevelopment by placental release of neurotransmitters including norepinephrine/epinephrine, dopamine, and serotonin, a relationship termed the “placenta-brain-axis” (Hodyl et al., 2017; Rosenfeld, 2021). Disturbances or pathophysiological shifts in placenta, and therefore *in utero* environment, can contribute to a variety of neurological disorders (Mir et al., 2021; Gardella et al., 2022; Parenti et al., 2022; Williams and LaSalle, 2022). For example, an analysis of the placental metabolome at birth following prenatal exposure to the teratogen phthalate revealed a correlation between the placental metabolome and ASD-associated neurodevelopmental outcomes in male, but not female, children (Parenti et al., 2022).

A controversial component of the placenta-brain axis is the possibility that the placenta also supports microbiota. Contrary to the dominant conceptual model that the uterus is free from pathogens (Blaser et al., 2021) and that the microbial colonization begins only during delivery, several studies have suggested the infant microbiome is initially colonized in fetal life, through vertical transmission by the uterus of maternal microbiota through the placenta, and subsequently amniotic fluid (Collado et al., 2016). Early studies demonstrated the presence of bacterial communities in the placenta and documented their impact on pregnancy outcomes and fetal health (Fardini et al., 2010; Stout et al., 2013; Aagaard et al., 2014; Collado et al., 2016). Placental colonization includes both commensal and pathogenic bacteria from the vaginotropic non-infectious microflora, oral cavity, and maternal intestinal lumen, likely *via* translocation through maternal circulation (Aagaard et al., 2014; Bangma et al., 2021). Pathogenic microbes’ invasion in the uterine environment, specifically within the placenta, has been linked with premature birth, epigenetic changes in the placenta and other tissues, and adverse neurological effects during fetal brain development and later in life (Goldenberg et al., 2000; Tomlinson et al., 2019, 2020). Microorganism invasion in the placenta has been suggested to disrupt fetal brain development through the activation of inflammatory pathways by bacteria-related pathogen associated molecular patterns and by changing placental function through epigenetic changes, such as placental DNA methylation (Tomlinson et al., 2019, 2020). However, it should be noted that the presence of fetal placental microbiota is not settled science, and some researchers have suggested that technical sources of contamination may have contributed to faulty study outcomes (Theis et al., 2019; Blaser et al., 2021; Sterpu et al., 2021). As the occurrence of placental microbiota is still controversial, additional studies in this field and analyses of placental microbial metabolites are required to fully understand the placental microbiota’s contribution to the placenta-brain axis.

### 2.4. Impact of maternal microbiota on neonatal colonization

In addition to the vertical transmission, i.e., direct colonization of the neonate’s gastrointestinal (GI) tract by maternal microbiota during delivery, environmental factors can significantly shape GI colonization during the neonatal period. These environmental factors include method of delivery, breastfeeding, prematurity, antibiotic exposure, maternal infection, and stress (Khan et al., 2016; Kalbermatter et al., 2021; Bolte et al., 2022; Miko et al., 2022). For example, vaginal deliveries transmit particular family-specific strains, and higher numbers of bacteria, to the neonate’s gut in comparison to cesarian section (Makino et al., 2013; Rutayisire et al., 2016; Kim et al., 2020). In a preclinical study on the impact of maternal stress on offspring microbiota and neurodevelopment, maternal stress decreased both vaginal *Lactobacillus* in pregnant mouse dams and decreased *Lactobacillus* colonization in the offspring (Jašarević et al., 2015). Additionally, the effects of early bacterial colonization of the neonate can be long-lived, even if the bacterial colonization is subsequently normalized. For instance, in one study, cesarean-delivered pathogen-free mouse pups were exposed to human vaginal microbiota by gastric gavage and compared to pups gavaged with microbe-free transport media (Jašarević et al., 2021). By adulthood the microbial population differences in treated and control offspring GI were no longer apparent, with similar measures of microbiome diversity and membership across groups. However, there were lasting impacts on the immune system and neural gene expression, as adult males had altered proportions of immune cells and hypothalamic gene expression across the neonatal microbial colonized and control groups. The data from this study indicate that alterations in the maternal environment not only shape their own microbiota, but also change neonatal colonization, which can have lasting consequences for offspring health.

### 2.5. Known role of gut microbiota in the etiology of neurodevelopmental disorders

Gut microbiota have been implicated in neurodevelopment and cognitive function. Studies have used microbial treatments and assessed the impact of microbiota alterations on behavioral outcomes. In a study examining the relationship between gut microbiota and memory formation, wild-type mice and germ-free mice were infected with pathogenic *Citrobacter rodentium* and cognitive performance was evaluated using the novel object recognition test and the T-maze in the presence or absence of acute stress (Gareau et al., 2011). Infection alone did not impair novel object exploration, but following acute stress, infected mice were unable to discriminate the novel object from the familiar object. This effect persisted 30d after infection by which time the active infection had been resolved. Similarly, active infection with acute stress decreased spontaneous alternation when exploring a T-maze. These stress-induced cognitive impairments were ameliorated with antibiotic treatment, indicating a causal link between gut microbiota and cognitive impairment. Gut microbiota have also been associated with anxiety behavior. Male mice were infected with pathogenic *Campylobacter jejuni* and evaluated for anxiety-like behaviors using a holeboard assay (Goehler et al., 2008). In the holeboard assay, the central middle hole, in an array of evenly spaced holes, is baited with a food reward. Each subject is evaluated for its time spent in the center of the assay, as well as the quantity of nose pokes into central vs. peripheral holes, with less time/nose pokes in the center indicating greater anxiety-like behavior. Mice with active infection spent significantly less time in the center and had fewer nose pokes in the center, baited hole. Anxiety-like behavior in infected mice was associated with aberrant regional cell activation, as demonstrated by c-Fos expression. Several stress-related brain regions were correlated with time in center (including basolateral amygdala and medial prefrontal cortex, among others) in non-infected mice, whereas a few brain regions largely associated with viscerosensory signal processing (bed nucleus of the striata terminalis, central amygdala, and nucleus of the solitary tract) were correlated with time spent in the center for infected mice.

Impacts of microbiota on anxiety-like behaviors may be transmissible from pregnant dams to their offspring. As previously mentioned, germ-free mice colonized with choline-consuming *Escherichia coli* have decreased DNA methylation (Romano et al., 2017). In this study, the researchers also examined germ-free pregnant mouse dams colonized with choline-consuming *Escherichia coli* and fed a high choline diet. Serum of colonized pregnant dams had lower levels of choline, while the fetuses had decreased DNA methylation in the brain but not in the liver. When a strain of knockout animals known to develop memory impairments and stereotyped behaviors (*Apoe*^−/−^) underwent the same treatment (colonized and fed a choline-supplemented diet), pregnant dams displayed elevated anxiety-like behaviors, including significantly increased rates of barbering and infanticide, compared to non-colonized pregnant dams. Moreover, offspring of the colonized pregnant dams had elevated rates of marble burying than the offspring of non-colonized dams, indicating increased anxiety-like behavior in the offspring. Several studies in rodent models have shown that probiotics can (a) improve cognitive function or prevent cognitive dysfunction, and (b) reduce stress-induced anxiety as well as depressive behaviors (Eastwood et al., 2021; Mindus et al., 2021; Natale et al., 2021). Collectively, these data emphasize a crucial role of gut microbiota in cognitive function and neurobehavioral outcomes, and the potential to target microbiota for therapeutic interventions.

Alterations to gut microbiota have also been implicated in neurodevelopmental disorders. Gut microbiome in children with ASD has been shown to be less diverse compared to children with neurotypical development (Kang et al., 2018). The authors of this study also hypothesized that a relationship may exist between altered fecal metabolites present in children with ASD, including increased levels of the neurotoxin isopropanol and decreased levels of the neurotransmitter γ-aminobutyric acid (GABA), which may contribute to altered neurodevelopment. This connection between gut microbiota and GABA signaling-related microbial metabolites is further supported in a preclinical study wherein germ-free (GF) mice were colonized with fecal microbiota from children with ASD or neurotypical development (TD) (Sharon et al., 2019). These colonized mice were then mated and offspring examined for neurobehavioral, microbial, and neural transcriptomic changes. In adolescence (postnatal day [PND] 45), offspring mice from ASD microbiota-colonized parents exhibited greater ASD-like behaviors than offspring mice from TD-colonized parents including increased repetitive behaviors, decreased vocalizations, and decreased social interaction, shown by the marble burying task, quantification of ultrasonic vocalizations, and the direct social interaction test, respectively. Not only could these offspring be separated by gut bacterial taxa, but the behavioral deficits were correlated with the expression of four species of bacteria. Using untargeted metabolomics analyses to determine the expression of microbial metabolites in the colon, the offspring from ASD microbiota-colonized mice had lower amounts of weak GABA receptor agonists 5-aminovaleric acid and taurine. However, while the expression of these agonists was not altered in serum, oral administration of 5-aminovaleric acid or taurine to the pregnant dam improved ASD-like behaviors in a separate ASD mouse model.

Attention-deficit/hyperactivity disorder (ADHD), is another neurodevelopmental disorder with a gut microbiota linkage. A systematic review of clinical literature found that, while numerous differences in microbiota have been reported, there were no overarching differences in microbiome diversity between children with ADHD and children with neurotypical development (Gkougka et al., 2022). In one study, *Faecalibacterium* content was inversely correlated with parental-reported ADHD severity in treatment-naïve children (Jiang et al., 2018). It should be noted that FASD and ADHD have a high co-morbidity. For example, a study that examined the association of adverse childhood experiences and neurodevelopmental disorders with FASD made an incidental observation regarding the comorbidity of ADHD and FASD (Kambeitz et al., 2019). In this study of individuals <22 years of age, seen at a regional FASD center, of those diagnosed with FASD, 85.7% were also diagnosed with ADHD, while 63.8% of those with suspected FASD, but nevertheless did not reach criteria for FASD diagnosis, were diagnosed with ADHD. It is possible that individuals who did not meet criteria for a FASD diagnosis may have sub-clinical impacts of PAE that are associated with increased rates of ADHD. Contrastingly, for 3–17-year-olds across the US, an estimated 9.6–9.8% have received an ADHD diagnosis (Bitsko et al., 2022). Notably, the neurobehavioral profile and brain structure alterations of PAE-associated ADHD are distinct from non-PAE-associated ADHD (Coles et al., 1997; Mattson et al., 2019; O'Neill et al., 2022) and, due to barriers in obtaining FASD diagnoses (Burd, 2016, see 4.1), these differences are not typically assessed. Therefore, the relationship between ADHD and microbiota is likely confounded by undiagnosed comorbid conditions, such as FASD, that can shape the profile of ADHD and, consequently, ADHD-related microbiome diversity.

There are overlaps in symptoms between neurological and neurodevelopmental diseases. ASD and ADHD are often co-morbid and share many common features, with one study at a tertiary center for ASD finding that 63% of ASD patients also displayed ADHD symptoms (Avni et al., 2018). Overlapping symptoms include anxiety, cognitive impairment, inattentiveness, and social difficulties (Avni et al., 2018). More importantly, ADHD and ASD patients both share common gastrointestinal disorders (Buie et al., 2010; Vargason et al., 2019) and for individuals with ASD, the severity of these gastrointestinal disorders positively correlate with the severity of the neurological disorders (McElhanon et al., 2014). Overall, many neurodevelopmental and neurological disorders appear to overlap both in symptoms and in the role of microbiota in shaping disorder outcomes and symptom severity.

## 3. Alcohol exposure affects maternal and infant gut microbiota

Although gut microbiota and their metabolites have been associated with neurodevelopmental disorders, we are currently just beginning to understand the relationship between PAE and gut microbiota in both the mother and fetus. Recent research found that ethanol exposure alters the plasma metabolite profile, both microbial and non-microbial, in pregnant mouse dams (Virdee et al., 2021). Principal component analysis of metabolites from ethanol-exposed and control exposure dams showed that metabolite profile well-segregated these groups. In particular, the first principal component segregated the alcohol-exposed and control dams, explaining ~23% of the overall variance between samples. In that study, microbial-derived metabolites were a major contributor to the separation between alcohol-exposed and control dams. Critically, microbial-derived metabolites were also found in fetal tissues, including the fetal brain. However, while microbial-derived metabolites in maternal plasma were promising biomarkers of ethanol exposure due to their ability to separate groups, microbial metabolites altered in fetal brain did not exhibit similar discriminatory power. Nevertheless, changes in fetal brain metabolites may have longer term consequences for neurodevelopment and need further investigation. Another preclinical study reported that PAE altered the fecal microbiota of adult offspring in a sex-specific manner (Bodnar et al., 2022). Pregnant Sprague–Dawley rats were fed ethanol containing liquid diet or a nutritionally matched chow throughout gestation (gestational day [GD] 1-GD 21).^1^ PAE offspring (at PND 80) exhibited more diverse microbial communities than control offspring. When stratified by offspring sex, this difference was found to be attributable to male, and not female, offspring. Intriguingly, in control offspring, males and females segregated in an analysis of microbial community composition while the PAE offspring, the microbial composition of males and female overlapped, suggesting that PAE may minimize sex differences. This study also showed that the effects of PAE on fecal microbiota are long-lasting and differences can be documented in adult offspring. The relevance of these preclinical findings are supported by a self-report survey by adults with FASDs, who reported intestinal complications (e.g., irritable bowel syndrome, chronic diarrhea, chronic constipation) at rates 1.8–2.5 times higher than the general population (Himmelreich et al., 2020), which can be attributed to altered gut microbial composition and function (Barbara et al., 2016).

The impact of alcohol exposure on gut microbiota has also been documented in individuals with alcohol use disorders (AUDs). Individuals with AUDs were found to have decreased bacterial community diversity and statistical analyses indicated these altered microbiota could be biomarkers of both amount of alcohol consumed and decreased cognitive function associated with alcohol consumption (Du et al., 2022). A systematic review of alterations to gut microbiota in individuals with AUD showed that a common outcome was a reduction in *Ruminococcus, Collinsella,* and *Prevotella* genera and an increase in Proteobacteria phylum (Litwinowicz et al., 2020). Anti-inflammatory and SCFA-producing species are decreased, which may contribute to decreased gut barrier function and increased inflammatory tone found in preclinical models of chronic alcohol use (reviewed in Leclercq et al., 2017). Moreover, as the above review noted, this inflammatory profile may further contribute to alcohol seeking behavior. Thus, several microbiota normalization strategies have recently been proposed and used as preventatives and therapeutics for AUDs by lowering systemic inflammation and restoring the gut barrier function. Fecal microbiota transplantation may be one method for treating AUDs by increasing beneficial microbiota (Bajaj et al., 2021). Bajaj and colleagues, in a double-blind randomized clinical trial, treated patients with AUD-associated cirrhosis and active AUD with fecal microbiota enriched in *Lachnospiraceae* and *Ruminococcaceae* or placebo. Fecal microbiota transfer decreased alcohol craving and increased microbiome diversity and plasma butyrate (a microbial-derived SCFA) levels in patients with AUDs 15 days after transplantation. Additionally, 6 months after the transfer, fewer AUD-related serious adverse events occurred in fecal microbial transfer patients compared to the placebo group.

For fetal microbial colonization, maternal diet is a major driver of microbiota composition (Miko et al., 2022). Alcohol exposure has been shown to drastically shape nutrition, including by altering both maternal and infant nutrient absorption which has been associated with neurodevelopmental delays in children with PAE.

## 4. Nutrition as a driver and consequence of PAE-altered microbiota

### 4.1. PAE-induced alterations to maternal and fetal nutrition

The consumption of recreational and illicit substances during pregnancy can contribute to nutrient malabsorption, especially micronutrients like zinc, choline, iron, copper, and selenium (May et al., 2016). This malnutrition has been associated with impaired GI absorption of nutrients in consumers, as well as drug-induced changes in food-intake preferences and behaviors, which contribute to unhealthy weight fluctuation and nutrient deficiency. Alcohol, particularly chronic alcohol consumption in adults has long been associated with malnutrition [for example, documented in early studies by Lieber (1975)]. Notably, changes in nutritional levels are directly associated with immune function, with poor nutrition serving as the leading cause of immunodeficiency worldwide (Chandra, 1997).

However, a body of literature conversely suggests that alcohol consumption can also stimulate food consumption (Caton et al., 2004, 2005) and that heavy consumption in particular is a risk factor for obesity (reviewed in Yeomans, 2010). Since deficiencies in essential nutrients can readily occur in individuals with obesity (Kobylinska et al., 2022), due in part to the consumption of calorie-dense foods low in essential nutrients, and to altered microbiota associated with obesity, this stimulation of food consumption may paradoxically lead to malnutrition. Eating patterns can, conversely, impact alcohol consumption behavior. For instance, dieters and mindful eaters surprisingly demonstrate greater instances of binge-drinking than non-dieters, specifically when dieting to achieve weight loss (reviewed in Caton et al., 2015). These effects may be further influenced by the physique and sex of drinkers (Colditz et al., 1991; Clevidence et al., 1995; Caton et al., 2007), as well as their age and racial identity (Wannamethee et al., 2004).

Ongoing studies in clinical and preclinical settings are investigating the effects of alcohol exposure during pregnancy on maternal and fetal nutritional profiles. In this section, we will highlight current literature describing the role of multiple common nutrients in either preventing or augmenting deficits associated with PAE.

#### 4.1.1. Metal ions

Metal ions, including iron, manganese and zinc among others, form core constituents of catalytic proteins and are critically important for maintaining cellular physiology including redox states in cells, but can also be toxic at high levels (Jomova et al., 2022). Therefore, destabilization of metal ion homeostasis may have significant consequences for tissue growth and toxicity.

##### 4.1.1.1. Iron

Preclinical studies have shown that PAE can impact fetal iron homeostasis, independent of maternal iron status. In a rat PAE model (Huebner et al., 2016), pregnant Long-Evans rats, following evidence of successful mating (GD 0.5) were provided iron sufficient (100 mg/kg) or deficient (<4 mg/kg) diets, and exposed either to 5 g/kg ethanol or isocaloric maltodextrin, via oral gavage, from GD 13.5 – GD 19.5. On GD 20.5, fetuses in the PAE-alone group had decreased body, liver, and brain weight. Following hematological assessment, the authors determined PAE disrupted iron distribution throughout the fetal body, sequestering iron in the liver with decreased iron in the brain. Although alcohol exposure did not affect overall maternal liver iron concentrations or transporters important for liver iron storage, alcohol exposure did significantly increase maternal gene expression of hepcidin. Hepcidin is a critical hormone for iron metabolism and elevated levels may reflect decreased ability for iron absorption in the gut. Notably, maternal iron levels were not predictive of fetal iron levels, and increased anemia observed in PAE offspring was not mediated by maternal iron status, suggesting that fetal iron absorption was directly inhibited by PAE. Further work in this model (Huebner et al., 2018), which examined an iron-fortified group (500 ppm) alongside the iron-sufficient group, found that PAE decreased fetal body weight, independent of maternal iron diet. In maternal and fetal subjects, PAE increased hepcidin levels beyond healthy levels (50–175 mcg/dL). When PAE was supplemented with an iron-fortified diet, hepcidin expression was normalized to non-PAE controls. Furthermore, hepcidin levels in PAE offspring corresponded to increased iron levels in the liver compared to maltodextrin controls, and increased iron levels in the brain compared to PAE without iron supplementation.

In a follow-up study, rat dams were fed an iron sufficient diet and further supplementation of iron through oral gavage (6 mg/kg, doubling daily intake) during the ethanol/maltodextrin exposure window had a positive impact on iron metabolism in PAE fetuses compared to vehicle-treated PAE fetuses (Helfrich et al., 2022). Compared to the maltodextrin-exposed controls, PAE fetuses without iron supplementation had decreased brain weight, body weight, and number of red blood cells, and increased levels of hepatic iron and rates of fetal anemia. With iron supplementation, PAE animals had no fetal anemia, normalized red blood cell counts, and increased brain weight, although only males experienced a recovery equivalent to non-exposed controls. These effects are conserved across species, as this group recently found that iron deficiency with concomitant PAE also reduces hemoglobin and red blood cells in a PAE mouse model (Helfrich et al., 2023). In this study, they further found that the underlying mechanisms for this change in hemoglobin are likely, surprisingly, changes in ribosome biogenesis and not hemoglobin synthesis or iron metabolism.

Clinical investigations have further validated the relationship between PAE and iron dysregulation (Bradley et al., 2022). Pregnant women in Cape Town, South Africa were recruited and categorized by their drinking patterns during the first trimester of pregnancy (Carter et al., 2007). If they reported two or more instances of binge drinking alcohol during pregnancy, or consumed ≥1.0 oz. of absolute alcohol (AA) per day, they were qualified as heavy drinkers. Light drinkers included women who averaged <1.0 oz. AA, the equivalent of 2 standard drinks, or reported ≤2 incidents of binge drinking (≥5 standard drinks per occasion). At 6.5 and 12 months of age, infant blood samples were collected to determine iron status. At 6.5 months, PAE was not associated with iron-deficiency anemia. However, PAE infants exhibited reduced birth weight and body length. In contrast, at 12 months of age, infants exposed to PAE in the binge drinking group experienced higher rates of iron-deficiency anemia diagnosis compared to abstinent/light drinking-exposed infants. Although PAE was associated with low infant weights independent of iron deficiency, iron levels moderated this relationship, with iron deficiency leading to the lowest birth weights among PAE offspring.

In the same population, the longitudinal impacts of iron deficiency were also examined (Carter et al., 2012). In this study, consumption of two drinks per day, or 4 drinks per occasion, was classified as “heavy drinking” while consumption of one drink per day without binging, or alcohol abstinence, was classified as abstaining/light-drinking controls. PAE was associated with lower offspring heights and weights at 6.5 months, 12 months, 5 years, and 9 years. Moreover, PAE was also associated with higher rates of iron deficiency across all age groups, with five-year-old children in the PAE group 6.5 times more likely to exhibit iron deficiency than the age-matched controls. A follow-up investigation in this cohort further determined that children with a history of PAE experienced different growth-deficit recoveries (Carter et al., 2016). Children born small for their age were classified as experiencing either catch-up growth, or long-term postnatal growth restriction, through the age of 13 years old. In children without a history of PAE, all but one child born small for their age (98.3%) experienced postnatal catch-up growth. This percentage was considerably lower for children born small for their age with PAE (58.5%). Furthermore, degree of gestational alcohol exposure corresponded with whether children experienced catch-up growth; specifically, children with the highest degree of PAE were the least likely to experience growth recovery. It is yet unknown whether iron deficiency contributes to and/or predicts growth trajectories in children with PAE throughout early life.

Decreased iron in offspring may be attributable, in part, to impaired maternal iron absorption, which has been implicated by elevated hepcidin levels in rodent models PAE (Huebner et al., 2016, 2018), as discussed above. In a separate investigation, pregnant women (*n* = 206) were interviewed during three prenatal visits, in which their drug-taking habits were recorded (Carter et al., 2021). Blood samples from infants (either 2 or 6.5 months old) and mothers, and tissue samples from corresponding placentas in control (non-drinking) and heavy-alcohol-drinking groups were later examined and compared. Heavy drinking during pregnancy was associated with increased maternal hepcidin, increased fetal iron sequestration in fetal blood samples, and decreased placental ferroportin and transferrin, which decreased maternal and neonate red blood cell synthesis (erythropoiesis). These restrictions in iron transport were associated with decreased fetal hemoglobin levels and increased iron-deficiency anemia diagnoses in 6.5-month-old infants. Maternal decreases in hemoglobin were positively correlated with neonatal hemoglobin deficits, which increased rates of infant iron-deficiency anemia.

Taken together, these studies indicate that PAE may impair maternal-fetal iron transportation across the placental barrier and result in persistent fetal iron deficiency and greater risk for childhood anemia. While iron supplementation serves as a promising therapeutic intervention for children with a history of PAE, it is important to note that negative side effects for iron supplementation have been reported, including constipation and bloating following oral administration, likely due to effects on gut microbiota (Bloor et al., 2021).

##### 4.1.1.2. Zinc

In a preclinical study (Keppen et al., 1985), pregnant CBA/J mouse dams on GD 1 were randomly assigned to treatment groups receiving liquid diet containing 15%, or 20% ethanol, or maltose dextran-containing isocaloric control from either GD 1–18 or GD 6–18. The liquid diet also contained low levels of zinc (0.3 μg zinc/ml) or a concentration of zinc that is the recommended dietary amount for reproduction in laboratory mice (8.5 μg zinc/ml). Low-zinc diet significantly reduced fetal weight on GD 18 compared to adequate zinc exposure. Low zinc with PAE (15 or 20%) co-exposure significantly increased fetal resorption (37–52%) compared to low zinc diet without PAE and adequate zinc diet with PAE (0–2%). With increasing levels of PAE, there were increased numbers of fetal malformations (e.g., precocious opening of eyelids, mild limb hypoplasia). This effect was most pronounced in the low-zinc/20% ethanol group where all fetuses exhibited malformations.

Prenatal alcohol exposure has additionally been associated with deficits in placental zinc transport (Ghishan et al., 1982). *In vivo* assessments of zinc transport revealed that PAE was associated with reduced zinc in the placenta and fetus, compared to pair-fed controls. In the first study, Sprague Dawley dams received ethanol (4 g/kg) via nasogastric intubation on GD 20 as a model of acute ethanol exposure. In the second study, Sprague Dawley dams received a liquid diet of either 5% ethanol or calorically-matched maltodextrin (controls) from GD 4-GD 20 as a model of chronic ethanol exposure. In both exposure paradigms, pregnant rats were anesthetized on GD 20 and administered radioisotope zinc-65 chloride injections (2 μCi/100 mg body weight) via an exposed femoral vein. Compared to control subjects, acute ethanol exposure reduced placental uptake of zinc-65 by 40%, while reducing fetal absorption by 30%, but did not impact overall placental and fetal tissue weights. In contrast, chronic ethanol exposure significantly decreased fetal weights at the time of tissue collection, while simultaneously increasing placental weights, compared to pair-fed controls. Similar to acute ethanol exposure, this chronic paradigm decreased zinc-65 placental uptake by 40% compared to controls. Ethanol-exposed dams also exhibited lower steady-state, non-radioactive zinc concentrations in their serum, although non-radioactive zinc levels in maternal femurs did not differ by exposure. Unsurprisingly, PAE fetuses also demonstrated significantly lower (−13%) zinc concentrations than alcohol-free controls, consistent with the reduction in placental uptake. This study importantly emphasizes that, even when mothers consume the daily recommended levels of zinc through diet, alcohol-exposed fetuses may require additional supplementation due to impaired placental transport.

Zinc deficiency has also been reported in children with a history of PAE. Infants with fetal alcohol syndrome (FAS, average 8.8 months old), a FASD diagnostic category characterized by facial anomalies, growth deficiencies including brain growth anomalies, and neurobehavioral impairment (Hoyme et al., 2016), had lower levels of zinc in their plasma than the control group, which contained slightly younger infants (average 6.9 months old) that were, nevertheless, on average, not significantly different from the FAS infants for height and weight (Assadi and Ziai, 1986). Yet, infants with FAS excreted significantly higher levels of zinc in urine than the control group, indicating that the decreased plasma levels of zinc may be due to increased excretion in the FAS infants.

##### 4.1.1.3. Selenium

Sufficient levels of selenium in pregnant individuals have been linked to healthy neurodevelopment of the neonatal cerebellum (Močenić et al., 2019). In contrast, deficient maternal selenium levels have been associated with increased risk of neurodevelopmental disorders, including ASD and ADHD (Lee et al., 2021). Selenium deficiency has previously been investigated in maternal and fetal tissues following gestational alcohol exposure (Ojeda et al., 2009). In this preclinical study, female Wistar rats were split into three experimental groups: control, alcohol, and pair-fed dams, who served as controls for alcohol-based malnutrition. Dams drank 20% ethanol *ad libitum* through the exposure paradigm, beginning 4 weeks before mating and continuing up to 21 days post-parturition. As established by many other models of FASD, PAE was associated with fewer viable pups/litter and lower body weights in PND 21 juveniles. In both the pair-fed and alcohol exposed groups, dams consumed less selenium, due to overall decreased dietary intake, and ethanol-exposed dams had decreased overall selenium balance, indicating increased excretion of selenium, compared to the pair-fed controls. These patterns were mimicked in offspring, with PAE offspring receiving less selenium from dams, but also eliminating more selenium than pair-fed controls. PAE-associated selenium deficits were specifically found within tissues of the liver, kidney, and heart in offspring, although future investigations of brain-specific changes in selenium would elucidate whether selenium malnutrition contributes to aberrant neurodevelopment in FASD.

#### 4.1.2. Other essential dietary nutrients

Vitamins A and E, the polyunsaturated fatty acid docosahexaenoic acid (DHA), and the micronutrient choline are only a few of the many nutrients essential for fetal development (Cortes-Albornoz et al., 2021). These nutrients support many cellular functions including: antioxidant capacity, cell membrane integrity, and one-carbon metabolism/methyl donor formation (Ducker and Rabinowitz, 2017; Blaner et al., 2021; Cortes-Albornoz et al., 2021). Therefore, undernutrition with regards to these nutrients can inhibit growth and development.

##### 4.1.2.1. Choline

Maternal choline insufficiency is associated with neural tube deficits while choline supplementation is associated with improved childhood cognitive function in infancy and potentially early childhood (Obeid et al., 2022). Preclinical, as well as clinical, research consistently shows that choline insufficiency increases risk of negative outcomes following PAE while choline supplementation promotes resiliency (for a systematic review see Akison et al., 2018). Additional research has further supported the use of choline as a therapeutic for PAE/FASD. In a rat model, the impact of choline insufficiency on the effects of PAE was investigated (Idrus et al., 2017). Pregnant Sprague–Dawley rats received diets with 40, 70%, or 100% of the daily recommended choline levels alongside exposure to ethanol (intragastric intubation, 6.0 g/kg/day) or two control groups, one that had caloric intake matched to that of the ethanol group (pair-fed) and one that was normally fed (*ad libitum*). These exposures occurred from GD 5 to GD 20. On PND 5–6, PAE was found to impair motor development, reduce body weight, and increase hyperactive behavior compared to both pair-fed and *ad libitum* groups. Notably, when PAE was concomitant with the highest choline deficit, 40% of the recommended daily intake, offspring experienced the most severe deficits in motor development and increases in activity compared to all other exposure groups.

Additional preclinical models have shown that choline supplementation can ameliorate PAE-induced negative outcomes. In a model of periconceptional alcohol exposure, Sprague–Dawley rats received 12.5% ethanol or 0% ethanol in liquid diet from 4 days pre-conception through GD 4. On GD 5, dams began a diet of either standard, 1.6 g choline/kg chow, or a choline-enriched diet of 2.6 g choline/kg chow (Steane et al., 2021, 2022). Fetal tissue samples were collected and analyzed on GD 20. Choline supplementation increased fetal liver weight and decreased fetal brain:liver ratio, a marker for fetal growth restriction (Westen et al., 2019), independent of alcohol exposure (Steane et al., 2021). Choline supplementation dose-dependently decreased placental and increased fetal plasma acetylcholine levels (Steane et al., 2022). Periconceptional alcohol co-exposure abrogated the impact of choline on acetylcholine levels for placentae of fetuses of both sexes and reduced acetylcholine levels in the plasma of female offspring only. Given that acetylcholine is one of the neurotransmitters in the placenta-brain axis (Rosenfeld, 2021), these findings indicate that periconceptional alcohol can interfere with this critical placenta-derived neurodevelopmental signaling.

The therapeutic potential of choline supplementation has been further demonstrated in clinical studies. Pregnant women who self-reported heavy alcohol consumption (time-line follow back, ≥2 standard drinks [1.0 oz. AA]/day, or ≥ 2 binge-drinking episodes) self-administered either 2 g packets of choline or placebo twice daily from 23 gestational weeks until birth (Jacobson et al., 2018; Warton et al., 2021). While PAE infants were small at birth, choline supplementation facilitated catch-up gains in weight and head circumference at 6.5 months and 12 months and increased the volume of brain regions, including the left and right thalamus, left and right caudate, right putamen, and corpus callosum. Moreover, the PAE infants with choline supplementation had improved performance on infant learning and memory tests, namely eyeblink conditioning and the Fagan test of infant intelligence.

A separate research group has similarly investigated the effects of choline supplementation on cognitive performance in children. A cohort of 60 children with FASD diagnoses (2.5–5 years old) were randomly assigned to receive 500 mg choline or a placebo daily for 9 months (Wozniak et al., 2015). Children received cognitive assessments prior to and following treatment, including an elicited imitation memory test, an assessment of explicit memory ability using hippocampal-supported behavioral imitation. Choline treatment was found to improve elicited imitation performance specifically in children under 4 years old, indicating a potential window of early development that is most beneficial for memory-recovery *via* choline treatment. In a follow-up study performed 4 years later, children were assessed on a multitude of cognitive and behavioral domains to look at long-term outcomes associated with the initial choline supplementation paradigm (Wozniak et al., 2020). Compared to children in the placebo group, children with FASD who received choline treatments exhibited greater aptitude for non-verbal visual spatial reasoning and working memory. The choline-receiving group also exhibited fewer behaviors associated with ADHD, according to parental self-reports. Unlike acute assessments in this cohort, the age of choline treatment did not affect overall outcomes in older children, indicating that long-term improvements were possible as long as children with FASD received choline between 2.5–5 years of age. To identify a possible mechanism underlying the benefits of choline supplementation on FASD symptomology, blood samples were collected from 52 children in this cohort and genotyped for ~400 single nucleotide polymorphisms (SNPs) associated with choline status and/or choline metabolism (Smith et al., 2021). Although baseline serum levels of choline did not differ between treatment groups, 14 SNPs associated with the choline transporter gene *SLC44A1*, were correlated with improved cognitive performance exclusively in children with FASD who received choline treatments. Although further research is needed, this may implicate a role for PAE in inhibiting proper choline transport, which is exacerbated by genetic risk factors, leading to FASD-associated cognitive impairment.

Although choline supplementation demonstrates promise for treatment in children with FASD, supplementation can also result in the adverse side effect of producing a fishy body odor. This effect can be common and increases in frequency with increasing doses of choline. In a randomized, placebo-controlled trial of choline supplementation, 56% of participants in the choline supplementation arm developed a fishy body odor that was noticeable during parental caregiving during the study, with 100% of participants in the top quartile of choline dose developing this body odor (Wozniak et al., 2013).

##### 4.1.2.2. Vitamin A/retinoic acid

Vitamin A and retinoids, the active metabolites of vitamin A, are required for appropriate embryonic development (Ross et al., 2000), including neurodevelopment (Haushalter et al., 2017). As some tissues are responsive to low levels of the most active retinoid, retinoic acid (RA, Ross et al., 2000), the use of retinoids is associated with teratogenic effects on fetal development. In these cases, fetuses were exposed to a vitamin A derivative 13-*cis*-retonic acid, the type commonly found in the treatment of cystic acne, psoriasis, skin diseases, or skin cancer (isotretinoin, brand name: Accutane), resulting in birth defects (Draghici et al., 2021). Notably, retinoic acid deficiency during pregnancy has also been associated with fetotoxic outcomes and birth deficiencies, including microphthalmia, respiratory distress, and abnormal cardiac, lung, and urogenital system function (Azaïs-Braesco and Pascal, 2000). However, at sub-teratogenic levels, vitamin A supplementation may protect against some PAE-associated negative outcomes. In one study, zebrafish embryos were exposed to ethanol (100 or 150 mM) during somitogenesis (2–24 h post fertilization [hpf]^2^) *via* the embryo medium (Muralidharan et al., 2015). At 48 hpf, embryos exposed to either ethanol concentration had microphthalmia, retinal lamination delay, and significantly decreased optic nerve width, compared to control counterparts. RA supplementation (1 nM, 2–24 hpf) ameliorated the effect of ethanol exposure on optic nerve width. Importantly, RA supplementation was less effective prior to RA biosynthesis period (2–16 hpf) compared to supplementation during RA biosynthesis (16–24 hpf), implicating an important window of development for improving supplementation treatment efficacy.

In a separate study, zebrafish were exposed to ethanol (150 mM) during a smaller early window of exposure, the period of gastrulation (5.25–10.75 hpf), with or without RA supplementation (1 nM) (Ferdous et al., 2017). At 2 days post fertilization, ethanol exposure significantly decreased zebrafish body length, an effect that was prevented by RA supplementation. RA supplementation improved the survival rates of ethanol-exposed embryos to the levels of the control exposure group. PAE during gastrulation also disrupted neuronal development, decreasing the cell body size, axon diameter, and frequency of synaptic response to excitatory innervation in Mauthner cells (M-cells) (Ferdous et al., 2017), a pair of neurons that mediate the escape response in fish (Pereda and Faber, 2011). RA supplementation prevented ethanol-induced decreases in cell body size and axon diameter and improved synaptic excitatory signaling, although not to control levels. Correspondingly, ethanol-induced increases in the angular velocity of the tail flip escape response, indicating an aberrant over-response, were prevented by RA supplementation.

The utility of vitamin A/retinoid supplementation to improve outcomes following PAE may be due, in part, to an amelioration of PAE-induced deficits in normally occurring retinol production. In a study of Sprague–Dawley rats (Kim et al., 2015), ethanol exposure altered the metabolism of vitamin A and its derivative retinoids, contributing to inappropriate retinol levels. Pregnant dams were fed a liquid diet of ethanol, an isocaloric liquid diet without ethanol (pair-fed), or access to typical rat chow. Ethanol-treated dams were given increasing percentages of ethanol from GD 7-GD 21 (2.2% on GD 7, 4.4% on GD 8, 6.7% on GD 9-GD 21). Independent of the exposure group, at PND 60–75, males demonstrated overall higher retinol levels than females. In males, PAE increased retinol levels in serum, while producing no significant effect in serum from female offspring. Additionally, PAE decreased retinol levels in male liver, lung, and prostate tissue compared to both control groups. In females, PAE increased retinol levels in liver and lung tissue compared to chow-fed controls, without producing a change in levels from pair-fed controls, suggesting this effect may be primarily driven by caloric intake. PAE lowered vitamin A (retinyl ester) levels in male and female lung tissues compared to pair-fed controls, without affecting the gene expression of the acyltransferase responsible for retinyl ester synthesis in mammals. These findings are particularly meaningful for potential PAE-induced effects on the microbiota, as the mammalian microbiota works symbiotically with the body to synthesize retinol from beta-carotene and prevent vitamin A deficiency (Srinivasan and Buys, 2019).

##### 4.1.2.3. Vitamin E

Vitamin E, which is exclusively available through diet, has well-established health benefits, including: antioxidant and anti-inflammatory actions, stabilization of cell membrane integrity, and promotion of immune function (Rizvi et al., 2014). Two preclinical studies in rat PAE models have demonstrated cognitive and metabolic benefits of vitamin E supplementation (Shirpoor et al., 2015; Mahdinia et al., 2021). In one of these studies, pregnant Wistar rats received 4 g/kg ethanol and a daily gavage of 100, 200, or 400 mg/kg vitamin E in sunflower oil from GD 0-PND 28 (Mahdinia et al., 2021). These perinatal ethanol exposed offspring were compared against two control groups that were not significantly different from one another and collapsed for analysis: an all treatment naïve group and a saline (ethanol vehicle) gavage group. Due to a lack of significant changes between an ethanol-alone exposure group and an ethanol with vitamin E vehicle (sunflower oil) control, these groups were also collapsed for analysis (ethanol-alone control group). At the end of this exposure period, offspring were tested on the Morris Water Maze for ethanol-induced deficits in memory and learning abilities. Perinatal ethanol exposure increased escape latency during the training, or learning, portion of the Morris Water Maze compared to controls, indicating diminished task learning. Vitamin E supplementation at both 100 and 400 mg/kg significantly decreased escape latency in ethanol-exposed rats compared to the ethanol-alone control group. During the probe trial, a test of memory, perinatal ethanol exposed offspring had significantly longer escape latency; concomitant 400 mg/dL vitamin E supplementation restored this impaired performance to non-PAE proficiency. An additional study that examined perinatal ethanol exposure (GD 7 – PND 21), during which pregnant Wistar rat dams were administered 4.5 g ethanol/kg and/or 300 mg of vitamin E by oral gavage (Shirpoor et al., 2015). In male offspring, PAE lowered body weight and disrupted lipid profiles at PND 21 and PND 90. Vitamin E supplementation during the period of ethanol exposure recovered ethanol-induced reductions in body weight and partially recovered lipid profiles. Perinatal ethanol-exposure also resulted in disrupted heart structure, with increased cellular proliferation in cardiac tissue and disrupted endothelial cell structure in the coronary artery, both of which were improved with concomitant vitamin E supplementation. Ethanol exposure also significantly elevated plasma pro-inflammatory markers, including cytokines. Vitamin E supplementation along with ethanol exposure significantly increased antioxidant capabilities in cardiac tissues; however, vitamin E was unable to fully ameliorate the impact of ethanol exposure on plasma pro-inflammatory cytokine levels.

##### 4.1.2.4. Docosahexaenoic acid

Docosahexaenoic acid (DHA) is an omega-3 (ω-3) fatty acid critical in the growth and functional development of the neonatal brain and deficits are well-associated with learning and memory difficulties (Richard and Calder, 2016). In a preclinical study of PAE in guinea pigs, researchers investigated how PAE affects fatty acid and phospholipid levels in the liver and brain (Burdge et al., 1997). Pregnant dams received either 6 g/kg/day ethanol, 0.5 g/kg/day of tuna oil (a nutritional supplement high in DHA), or a combination of both, for 2 weeks prior to and throughout pregnancy. Phosphatidylcholine (PC) and phosphatidylethanolamine (PE) levels in the liver and brain were quantified in exposed offspring. In several cases, tuna oil supplementation recovered fatty acid levels in PAE offspring to the levels of non-exposed controls. In the fetal brain, ethanol exposure alone significantly reduced stearic acid and DHA fatty acid levels, which were completely recovered in PAE offspring with DHA supplementation. Furthermore, PAE increased the ratio of overall brain PC:PE levels from control animals, a ratio that was re-established with the addition of tuna oil treatment. In the fetal liver, PAE was significantly associated with reductions in DHA, and increased levels of ω-6 fatty acids, namely γ-linoleic acid, arachidonic acid, and docosapentanoic acid, all of which were recovered when alcohol exposure was combined with DHA administration. These data highlight a possible correlation between ethanol exposure and the biosynthesis of brain phospholipids PC and PE, which may underly cognitive challenges experienced by individuals with FASD.

DHA supplementation can also improve PAE-induced deficits in social behavior and somatosensory performance (Wellmann et al., 2015). In this study, pregnant Long-Evans rat dams were assigned to one of three exposure groups: ethanol exposure or pair-fed controls, both of which received a liquid diet from GD 6 – GD 21, or an *ad libitum* chow control group. For ethanol-exposed dams, ethanol concentrations in the liquid diet were escalated from 2.1 to 6.3% for GD 11- GD 21. Later, male offspring (PND 11 – PND 20) were randomly assigned to receive either artificial milk alone or supplemented with 10 g/kg DHA, or received no treatment as an additional control. Along with other reported deficits, PAE offspring, compared to pair-fed and chow controls, engaged in significantly less play fighting during adolescence, had fewer ultrasonic vocalizations in response to social isolation on PND 14, and had worse somatosensory performance on a gap crossing test, with PAE offspring crossing shorter gaps. Importantly, these deficits in play, vocalizations, and somatosensory performance were recovered by postnatal DHA administration, supporting the role of DHA in facilitating healthy offspring behavior.

### 4.2. The reciprocal relationship between nutrition and microbiota

As ongoing research continues to explore the relationship between PAE and malnutrition, it is important for scientists and clinical practitioners to consider how nutrient imbalance affects microbiota function in pregnant individuals and their offspring. Dysregulated choline levels, for instance, contribute to unhealthy levels of trimethylamine-*N*-oxide in serum, which are associated with the onset of non-alcoholic steatohepatitis, cardiovascular disease, and chronic kidney disease (Arias et al., 2020). Gut bacteria rely on iron stores to replicate and survive; however, iron over-abundance in the gut produces reactive oxygen species, inducing oxidative stress and intestinal damage (Yilmaz and Li, 2018). The retinoic acid derived from vitamin A consumption stimulates immune cell migration to the intestines and maintains the health of the gut barrier (Iyer and Vaishnava, 2019). Vitamin A deficiency, in contrast, impairs the immune response to intestinal challenge, and can increase risk for colorectal cancer (Jijon et al., 2018). Changes in vitamin A, zinc and vitamin E levels are each associated with altered gut bacteria composition (Chen et al., 2020; Choi et al., 2020; Skalny et al., 2021), while selenium deficiency contributes to a gut environment that is more susceptible to thyroid, inflammatory, and cardiovascular dysfunction (Ferreira et al., 2021). Finally, DHA supplementation in mice was recently associated with reduced adipose inflammation and the reversal of insulin resistance following consumption of a high-fat diet (Zhuang et al., 2020). Thus, future research into PAE-induced changes in the microbiota would benefit from investigating nutrient levels as a moderating factor in alcohol-associated changes in gut-microbiota associated health outcomes.

## 5. Potential contribution of microbiota plasma biomarkers for FASDs

### 5.1. Plasma markers of PAE vulnerability

Fetal alcohol spectrum disorders are difficult to diagnose, as they require a team of physicians/pediatricians with specialized training (Jones et al., 2006; Burd, 2016; Bernes et al., 2021). Moreover, for diagnosis by clinicians, we are woefully under capacity, with the estimated ability to diagnose only ~1% of FASD cases worldwide (Burd and Popova, 2019). Critically, while metabolite biomarkers of ethanol can be used to assay PAE, they are limited by the time frame of exposure they can reflect and can be difficult to collect, e.g., meconium collection to assay fatty acid ethyl esters (Bakhireva and Savage, 2011). Research by our group and others have identified biomarkers in plasma that are able to risk stratify offspring and indicate vulnerability for future FASD development and/or neurodevelopmental delay. However, the origin of these biomarkers is currently unknown. Altered gut (and potentially placenta) microbiota likely contribute to and shape the profile of plasma biomarkers.

### 5.2. Extracellular vesicles

Extracellular vesicles (EVs) are membrane-bound vesicles that are released by cells and carry nucleic acids, proteins, lipids, and metabolites (van Niel et al., 2018). EVs that are released from one cell can fuse with, and release cargo into, other cells and impact the recipient cell’s physiology as a form of endocrine signaling. Work in adults who consume alcohol has shown that EVs can be part of the endocrine signaling of alcohol use, including increased inflammatory tone. In healthy adults, binge alcohol consumption increases the amount of a sub-type of EVs, namely exosomes, present in blood serum (Momen-Heravi et al., 2015). In the same study, serum exosome number was also elevated in mice following binge-like ethanol exposure. Moreover, the levels of microRNA (miRNA) cargo, miR-122, was increased in exosomes following alcohol exposure in both systems, and this elevation was found in culture to reprogram monocyte cells resulting in aberrant elevation of proinflammatory cytokines following immune challenge. Along with miR-122, additional EV cargo has been identified as potential biomarkers for alcohol-associated liver damage and disease (Cho et al., 2018).

Altered EV dynamics and cargo have been implicated in diseases with developmental origins, for which prenatal exposure to stress and drugs of abuse, including PAE, may act as an “initial hit” to promote the development of these diseases (Pinson et al., 2021). In preclinical models, PAE has been shown to alter EVs in the developing brain. In an *in vitro* neural stem cell culture model, ethanol exposure altered neural stem cell-derived EV cargo (Tseng et al., 2019a; Pinson et al., 2022) and proteome (Chung et al., 2022). These alterations to EV cargo can have functional consequences for recipient cells. When ethanol-naïve neural stem cells were exposed to EVs from other ethanol-naïve neural stem cells, the recipient neural stem cells had significantly decreased oxidative metabolism and proportion of cells in S-phase (Chung et al., 2022). However, when EVs were added that were derived from stem cells exposed to 320 mg/dl ethanol, these effects on ethanol-naïve neural stem cell metabolism and cell cycle were decreased, suggesting that changes in EV composition following PAE have a biologically relevant impact for the recipient cells. Similarly, hypothalamic microglia-derived EVs have altered cargo following ethanol exposure in a rat early postnatal/third trimester-equivalent exposure model (Mukherjee et al., 2020). These microglia-derived EVs contributed to the PAE-induced death of hypothalamic β-endorphin neurons *in vivo* and *in vitro*, potentially through increased complement-protein induced apoptosis.

Clinically, a subset of EVs in maternal circulation, carrying markers that were interpreted as suggestive of fetal origin, were found to carry potential biomarkers of early deficits associated with PAE (Darbinian et al., 2022). Within these potential fetally-derived EVs, molecules associated with brain development, including proteins myelin basic protein and synaptic protein synapsin-2 and miRNA miR-9, were significantly correlated with eye size, and therefore could be biomarkers of PAE-induced growth deficits (see 5.3 for more about miRNA biomarkers). Preclinically, in a rat model of PAE, amniotic fluid EVs had altered miRNA content following PAE (Tavanasefat et al., 2020). *In vitro*, these PAE-altered EVs had functional consequences for stem cell development, reprogramming bone marrow stem cells to reduce osteogenic differentiation. The effects of the PAE-altered EVs on stem cells were similar to effects seen following low dose ethanol exposure, indicating that PAE may directly and indirectly, through EVs, impact stem cell biology *in vivo*. This anti-osteogenic effect was attributed to the upregulation of three miRNAs within the EVs, as overexpression of these miRNAs in the bone marrow stem cells was sufficient to decrease pro-osteogenic RNA transcript expression. These amniotic fluid EVs may also play a key role in gut development, as amniotic fluid is digested by the fetus and provides trophic factors for intestinal growth (Dasgupta et al., 2016). Amniotic fluid may also be the primary source for fetal microbial colonization of the intestinal tract (e.g., Stinson et al., 2019; He et al., 2020), although this route of colonization is debated (Perez-Munoz et al., 2017).

Microbiota, including both gram-positive and gram-negative bacteria, can also release EVs. These microbiota EVs are taken up by intestinal epithelial cells and their cargo can: impact barrier function; promote cytokine release by epithelial cells; and be repackaged into epithelial cell EVs and released into host circulation (reviewed in Kaparakis-Liaskos and Ferrero, 2015). For example, in an *in vitro* model of the gut endothelial barrier, EVs, but not cell lysates, from probiotic, gram-negative *Escherichia coli*, stimulated cytokine release from intestinal epithelial cells (Fábrega et al., 2016). These endothelial cells were in a co-culture system that also contained peripheral blood mononuclear cells (PBMCs), which were spatially segregated from the endothelial cells. The EV-stimulated cytokines released by the endothelial cells induced TNFα expression in PBMCs. In disorders with intestinal barrier disruption, microbiota-EVs can also be released into circulation. Clinical subjects with disrupted endothelial barrier function show higher levels of microbiota-derived EVs, with more plasma EVs containing lipopolysaccharide (LPS) (Tulkens et al., 2020). In these subjects, the amount of LPS activity in the EVs was positively correlated with a plasma marker of increased intestinal permeability. LPS was also detected in EVs from some control subjects, albeit at lower levels, indicating that additional mechanisms may exist to allow microbiota EVs into circulation, including sub-chronic or transient gut dysbiosis. These microbial EVs can stimulate immune response; however, when derived from probiotic bacterial strains, they may also be beneficial. In a mouse model of colitis, in which intestinal inflammation is induced by administration of dextran sodium sulfate (DSS), the profile of bacterial contributors to the fecal EV milieu was altered to a greater degree than the change seen in microbial taxa diversity in the DSS animals (Kang et al., 2013). For the strain *Akkermansia muciniphila*, DSS did not alter the representation of *A. muciniphila* in the gut flora but significantly reduced the amount of fecal EVs from *A. muciniphila*. Oral administration of *A. muciniphila* EVs alongside DSS was able to recover DSS-induced weight loss, damage to colon epithelial cells, and inflammatory cell infiltration in the colon. None of these symptoms were improved when *A. muciniphila* bacteria was co-administered with DSS.

As briefly mentioned, microbiota-derived EVs and EVs released from endothelial cells containing microbiota-derived EV cargo play a role in the gut-brain axis, in part, by modulating inflammation (reviewed in Cuesta et al., 2021). Previous work indicates that cytokines, a key modulator of inflammation, are not only altered by PAE but can also serve as biomarkers of prenatal exposure and child outcomes. For example, in a clinical study, cytokines were assessed in maternal plasma during the second and third trimesters (Bodnar et al., 2018). Not only were individual cytokines altered by maternal alcohol exposure status, but altered cytokines were also associated with future offspring neurocognitive outcomes, i.e., whether a child showed neurodevelopmental delay. Moreover, statistical modeling identified unique groups, or networks, of cytokines that were associated with alcohol-dependent or alcohol-independent offspring neurodevelopmental delay, indicating that cytokines could discriminate PAE effects from other sources of neurodevelopmental delay. Similarly, in the offspring, networks of plasma cytokines were also able to indicate previous PAE as well as neurodevelopmental status (Bodnar et al., 2020). Preclinically, a number of factors have been shown to shape these cytokine profiles including additional endocrine molecules (Bodnar et al., 2016), environmental factors like early life stress (Raineki et al., 2017), and genetic sex (Raineki et al., 2017). Given the known roles of gut microbiota, both through EVs and other mechanisms, to shape host inflammatory tone and cytokine profiles (Mendes et al., 2019), further work needs to be done to understand the contribution of gut microbiota to PAE-altered cytokines.

A few preclinical studies have indicated that microbiota-derived EVs can cross the blood–brain barrier. For example, two studies using *in vivo* fluorescent computed tomography imaging of mice found labeled EVs from *Escherichia coli* (Jang et al., 2015) and *Staphylococcus aureus* (Choi et al., 2015) in the mouse heads, although the overall distribution within the brain was not assessed. A similar study that examined *Heliobacter pylori*-derived EVs did not find similar localization to the head (Choi et al., 2017), suggesting that trafficking to the head, and potential subsequent crossing of the blood–brain barrier, by these microbial EVs may be species-of-origin specific. One study examined the brain distribution of *Aggregatibacter actinomycetemcomitans*-derived EVs as a potential link between periodontitis and neuroinflammation (Han et al., 2019). *A. actinomycetemcomitans* EVs with fluorescently labelled membrane and RNA cargo were injected into the heart of 6-week-old male mice. Acutely following the injection (4 h), the EVs were localized within brain blood vessels. After 24 h, diffuse fluorescent signal for both the EV membrane label and the RNA cargo label was found in brain parenchyma, indicating that the microbiota-derived EVs crossed the blood brain barrier and released their RNA cargo.

While microbiota-derived EVs have not been studied in the context of PAE, these data suggest that microbiota likely shape host EVs, including their miRNA and long non-protein-coding RNA (lncRNA) cargo. Critically, these non-protein-coding RNAs have also been implicated in the etiology of FASDs.

### 5.3. Non-protein-coding RNAs

Non-protein-coding RNAs (ncRNAs) are RNAs that are transcribed but not translated into proteins (Mattick and Makunin, 2006). ncRNAs include multiple RNAs that can range in size, from small, e.g., miRNAs that are 18–22 nucleotides in length, to large, e.g., lncRNAs that are >200 nucleotides in length. These ncRNAs are multi-functional and impact developmental biology, including stem cell biology (Tseng et al., 2018), and are implicated in PAE and FASD (Mahnke et al., 2018). Microbiota can shape both peripheral and brain expression of miRNAs and lncRNAs, indicating a critical role for microbiota in ncRNA biology.

#### 5.3.1. miRNAs

miRNAs act within the cell to fine-tune translation (Bushati and Cohen, 2007), and in the nucleus to control transcription (Roberts, 2014). miRNAs can be released from cells into extracellular fluids, including blood plasma, where they can act as endocrine molecules. We (Balaraman et al., 2016; Salem et al., 2020), and others (Gardiner et al., 2016), found that alcohol exposure during pregnancy can alter the circulating miRNA profile, i.e., miRNAs present in blood plasma and serum. Plasma miRNAs in pregnant women were found to be indicative of future child outcomes (Balaraman et al., 2016; Tseng et al., 2019b), including growth restriction and neurodevelopmental delay. Moreover, in a preclinical mouse model, tail vein injection of these human-identified predictive miRNAs resulted in decreased fetal and placental growth in the absence of ethanol exposure (Tseng et al., 2019b). These data indicate that circulating miRNAs can be biomarkers of effect following PAE as well as endocrine mediators of negative health outcomes, including growth deficits.

Plasma miRNAs present during the neonatal and early infancy periods can also predict future health outcomes. miRNAs in umbilical cord plasma were found to predict neonatal opioid withdrawal syndrome development and severity in neonates prenatally exposed to medications for opioid use disorders (Mahnke et al., 2022). These withdrawal symptoms can develop from 24 h to 7 days after birth (Hudak et al., 2012); indicating that plasma miRNAs can predict short-term neonatal outcomes. Similarly, infant plasma miRNA profile at 2 weeks and 6.5 months of age was altered by PAE (Mahnke et al., 2021). Groups of PAE-altered miRNAs, which were likely biologically related, were identified. The groups of miRNAs in each age group that were associated with inflammation were found to statistically mediate PAE-induced growth deficits and neurodevelopmental delay, indicating that the variance in expression of these miRNAs is related to the PAE-associated outcomes. These data suggest that miRNAs have the potential to predict longer term changes that arise from prenatal exposures. It is likely that a number of tissues secrete miRNAs that contribute to the overall plasma miRNA profile, and gut microbiota are key regulators of the intestinal contribution.

Appropriate miRNA expression in intestinal epithelial cells is critical for intestinal barrier function. Mice with intestinal epithelial cell specific knockout of *Dicer1*, an obligatory gene for the generation of miRNAs, have substantially decreased miRNA content, aberrant intestinal epithelium structure, increased trans-epithelium transport, disrupted expression of genes associated with immune-related pathways, and increased invasion of immune cells into the lamina propria (McKenna et al., 2010). An additional study in intestinal endothelial cell *Dicer1* knockout (KO) mice found that miRNAs had predominantly decreased fecal expression compared to *Dicer1*-expressing control animals (Liu et al., 2016). The *Dicer1* KO mice also had greater between-mouse microbiome diversity and Proteobacteria as the dominant phyla. In contrast, *Dicer1-*expressing mice that had greater between-mouse microbiota similarity and Firmicutes as the dominant phyla. Fecal miRNA can: shape microbiota profile, as fecal miRNA from *Dicer1* expressing mice shifted the microbiome diversity in *Dicer1* KO mice to a higher similarity/lower diversity; and alter intestinal barrier function, as fecal miRNAs from *Dicer1* expressing mice ameliorated weight loss and colon damage in *Dicer1* KO animals in response to DSS-induced colitis. Additional research demonstrated that microbiota induce miRNA expression in intestinal epithelial cells, and thereby regulate gut barrier permeability. In this study, intestinal epithelial cells from conventional mice were found to have 1.5- to 3-fold higher levels of miR-21-5p than germ-free mice, indicating that microbiota regulate intestinal epithelial cell miRNA expression (Nakata et al., 2017). *In vitro*, the elevation of miR-21-5p was implicated with increased barrier permeability, as inhibition of miR-21-5p expression in intestinal epithelial cells increased intestinal barrier electrical resistance, indicating decreased permeability. The researchers further found that miR-21-5p inhibited expression of second messenger signaling pathway members, leading to increased expression of ARF4 which suppressed tight junction proteins.

The relationship between microbiota and miRNAs is not limited to intestinal epithelial cells. Altered circulating miRNAs and gut microbiota were found in human subjects with body mass index in the “obesity” range compared to subjects in the “normal” range (Assmann et al., 2020). A subset of the altered miRNAs and microbiota were correlated across samples, including miRNAs with mRNA targets that include pathways associated with mineral and vitamin absorption. These data suggest a relationship between circulating miRNAs and the microbiota that could be direct, e.g., circulating miRNAs can be in the gut lumen and directly alter microbiota, or indirect, e.g., similar biological mechanisms contribute both to the altered miRNAs and microbiota, which could include responses to altered adiponectin or insulin levels. It is also known that microbiota can shape circulating miRNA profile (Monaghan et al., 2021), which could also explain the correlation between circulating miRNAs and microbiota in these patients.

#### 5.3.2. Long non-coding RNAs

lncRNAs have multiple functions within the cell, including regulating transcription, translation, chromatin remodeling, and protein activity (Mahnke et al., 2018; Sanchez Calle et al., 2018). lncRNAs are important for neurodevelopment, including neurogenesis, cell proliferation, and synaptic pruning (Arzua et al., 2021).

Neural lncRNAs are altered following PAE. In murine neural stem cells, expression of *Oct4pg9*, a lncRNA that arose from a gene duplication event of the pluripotency gene *Oct4*, is increased by ethanol exposure (Salem et al., 2021b). The increased expression of *Oct4pg9* was associated with decreased expression of *Oct4*, which may reflect a reciprocal relationship between the parent gene and the lncRNA as single cell RNA sequencing revealed that *Oct4* and *Oct4pg9* were only co-expressed in ~2% of cells expressing either transcript. In the early murine developing neocortex, single cell RNA sequencing showed that ethanol-exposed female fetuses had decreased X-chromosome inactivation, with decreased expression of the lncRNA *Xist*, increased expression of the reciprocally regulatory lncRNA *Tsix*, and increased expression of genes on the X-chromosome that are typically within the inactivation regions (Salem et al., 2021a). Additional studies using post-mortem tissues of patient with AUDs also indicate that alcohol can impact lncRNAs as a mechanism to disrupt cellular function (reviewed in Mahnke et al., 2018).

Emerging evidence supports a role for intracellular epithelial cell lncRNAs in regulating intestinal epithelium integrity (reviewed in Xiao et al., 2021). The lncRNA *H19* can regulate epithelial health and recovery. In a mouse model of intestinal inflammation, the expression of *H19* was increased in intestinal epithelial cells following systemic administration of LPS (Geng et al., 2018). In genetically modified mice, deletion of the first exon of *H19* impaired crypt epithelial cell proliferation and impaired intestinal recovery following colitis. Moreover, intestinal cell lncRNA content is affected by microbial content. In a comparison between conventional mice, which have commensal gut bacteria, and germ-free mice, which are not exposed to microorganisms, conventional mice had >900 lncRNAs differentially expressed compared to germ-free mice in intestinal tissues (Dempsey et al., 2018). Furthermore, intestinal cell lncRNA content can be shaped in a microbial strain-specific manner. Existing intestinal transcriptome data (Joyce et al., 2014) were reanalyzed to compare lncRNA content between germ-free mice and germ-free mice that were colonized with either commensal microbiota or *Escherichia coli* with or without bile salt hydrolase (Liang et al., 2015). Colonization of germ-free mice upregulated 612 lncRNAs, but only 70 of these lncRNAs were upregulated in more than one group, indicating strain-specific modulation of intestinal lncRNA expression. Intriguingly, microbial colonization of germ-free mice altered lncRNA expression in other organs, including immune related organs (Liang et al., 2015), adipose tissue (Dempsey et al., 2018), liver (Liang et al., 2015; Dempsey et al., 2018), and, critically, the hippocampus (Liang et al., 2015). Additional research has implicated lncRNAs in neurological impacts of altered microbiota. *In silico* analysis of existing mouse transcriptomics data sets found that *Pten* and *Vegfa* were implicated as hub genes for altered gene expression in both the hippocampus and ileum in chronic stress-induced depression models (Abedpoor et al., 2022). Four lncRNAs were identified that were associated with both *Pten* and *Vegfa* and three of these lncRNAs (*Hotair*, *Meg3*, *Gas5*) had similarly altered expression in the hippocampus and ileum of depressed mice. Following an intervention of exercise and leucine, which ameliorated depression-related behaviors, the expression of *Pten, Vegfa*, and the three lncRNAs were partially recovered in both the hippocampus and ileum. In an additional study, germ-free mice were colonized with microbiota from patients with major depressive disorder or contrast patients without depression (Liu et al., 2021). Following fecal transplant, two mRNA-lncRNA-miRNA gene networks were identified in the hippocampus that were significantly different between the two groups. Both of these studies identified hippocampal ncRNA/mRNA networks associated with the gut-brain axis in depression.

One potential contributor to microbiota-altered lncRNA expression across tissues is microbial EVs. Bacteria create small RNAs (sRNAs) that range from 70 and 500 nucleotides in length (Gomez-Lozano et al., 2014), a size range that partially overlaps that for lncRNAs. These sRNAs can be packaged into microbial EVs and affect recipient cell function (Koeppen et al., 2016). As EVs can be found in blood circulation following intestinal barrier disruption, these lncRNA-like cargo may regulate cell biology throughout the host in a similar manner to the host’s lncRNAs.

## 6. Microbial contribution to risks for later-in-life health outcomes

The gut microbiota that is established very early during a newborn’s life has a significant and long-lasting impact on the overall health and well-being of the individual. A healthy gut microbiota provides a good start for the offspring’s metabolism and immune system that can reduce the risk of adverse secondary conditions later in life. Disruption of the gut by PAE may be part of an initial hit that can lead to increased risk for secondary conditions throughout the lifespan for individuals with FASDs. Preclinically, in an PAE rat model, increased amounts of inflammatory cytokines were found in the mesenteric adipose tissue of adult offspring (Bake et al., 2021) which could be due, in part, to altered microbiota and/or intestinal barrier function. There are also known reciprocal relationships between gut microbiota and metabolic disease (Cani and Delzenne, 2009), and there is clinical evidence for higher rates of multiple metabolic abnormalities in males and an increased risk of being overweight/obese in females with FASDs (Weeks et al., 2020). In this study, effects were conserved across species, as male zebrafish exposed to ethanol embryonically were also predisposed to metabolic disorders and altered visceral adipose content. In a rat model, adult PAE animals experience worse acute stroke outcomes and impaired long-term recovery following an ischemic stroke, with evidence for increased gut permeability following stroke (Bake et al., 2022). Recent work has shown that gut health is an important therapeutic focus for stroke recovery, as intestinal epithelial stem cell transplant has been shown to improve both acute and long-term recovery following ischemic stroke in a rat model (Mani et al., 2023).

Therefore, early interventions targeting microbiota could lead to improvements not only in neurodevelopment, but health across the lifespan following PAE.

## 7. Probiotics/prebiotics as a potential therapeutic approach for FASD

As nutritional interventions can directly impact gut microbiota, and nutritional interventions have been efficacious in decreasing negative outcomes following PAE (see section 4), the question becomes whether directly targeting gut microbial communities can have similar positive impacts following PAE. Furthermore, can interventions targeting gut health improve outcomes for secondary conditions which have worse outcomes following PAE, such as stroke, metabolic disorders, and cancer (Burd et al., 2014; Upreti et al., 2022)? In medical and clinical contexts, manipulation of gut microbiota is achieved through the use of pre-, pro-, and syn-biotics. Probiotics contain live microorganisms, whereas prebiotics containing nutritional components (usually non-digestible food fibers) that shape the gut microbial profile and synbiotics contain both probiotics and prebiotics (Pandey et al., 2015). As only a few studies to date have investigated the impact of PAE on gut microbiota, it is yet unknown whether pre/pro/synbiotics could be used as a therapeutic strategy for FASD. However, the administration of the pre/post/synbiotics has been shown preclinically to improve outcomes in neurobehavioral and neurodegenerative disorders, including depression, stress and anxiety-like behaviors (Bravo et al., 2011; Abildgaard et al., 2017), Alzheimer’s disease (Rezaei Asl et al., 2019), Parkinson’s disease (Rocha et al., 2015). Notably, preclinical research also shows these therapies can improve outcomes in neurodevelopmental disorders, such as ASD (Rocha et al., 2015; Siniscalco et al., 2018). In a randomized, double-blind, placebo-controlled clinical study, *Lactobacillus plantarum 299v* was used as a probiotic bacteria to enhance cognitive functioning in depressed patients (Rudzki et al., 2019). Compared to the placebo group, there was a significant decrease in kynurenine concentration, a tryptophan metabolite made in the liver that negatively affects mood and cognition, in the probiotic-treatment group. Another randomized controlled trial (Santocchi et al., 2020) used a patented and marketed multiple probiotic mixture, Vivomixx®, to investigate the efficacy of probiotic therapy for ASDs. Preschoolers with ASDs were administered either Vivomixx® or placebo based on their daily weight for 6 months. While probiotic supplementation had no statistically significant impact on the severity of ASD in the overall sample, an exploratory secondary analysis compared outcomes in children with ASDs between those who did and did not experience GI symptoms. Probiotic treatment significantly reduced the severity of ASDs in children without GI symptoms, and improved some GI symptoms, adaptive functioning, and sensory profiles in children with GI symptoms.

Given the growing body of evidence for the contribution of maternal-fetal microbiota to neurodevelopment, more research is needed to learn how PAE affects the microbiota, and how pre/pro/synbiotics can be used as a therapeutic approach for FASDs.

## 8. Conclusion and future directions

The gut microbiota is likely a critical contributor to negative outcomes following PAE, particularly with self-reported rates of gut disturbances in adults with FASDs (Himmelreich et al., 2020) and recent preclinical studies showing dysbiosis in PAE offspring (Bodnar et al., 2022) as well as alterations to bacterial metabolites in the maternal and fetal compartments following alcohol exposure (Virdee et al., 2021). More research is needed to understand how PAE may impact systemic function through the microbiota, including investigation of microbial contribution to biomarkers present in circulation, and how these microbiota impacts may differ by age, genetic, sex, and other key biological variables. Research of other neurodevelopmental disorders has implicated microbiota as a contributing factor to symptom development and severity and have demonstrated promising efficacy of microbial-based interventions. There is likely a reciprocal relationship between the nutritional deficits found following PAE and alterations to the gut microbiota, indicating that probiotic and prebiotic therapies could ameliorate negative outcomes following PAE and improve the quality of life for individuals with FASDs.

Notably, many of the studies implicating the microbiota and nutrition in FASDs and other neurodevelopmental diseases are correlational. In addition to the needed experiments and gaps in knowledge highlighted throughout this review, further experiments are required to interrogate where microbiota and nutrition are mechanistically linked to FASDs and determine systemic locations for therapeutic intervention. Rigorous experiments should be designed to account for subject variables that influence microbiota composition, including chromosomal and gonadal sex, diet, medication usage, age, and, in animal research, species. Gender, or the ways the presentation of genetic sex is impacted by sociocultural factors, is likely also a critical variable, particularly as receipt of gender-affirming care has been shown to alter the microbiome (reviewed in Krakowsky et al., 2022), but is drastically understudied. Sex is a particularly important factor for consideration, as sex-specific microbiotas have been identified in human and animal research, and has been the subject of recent review articles (Kim and Benayoun, 2020; Valeri and Endres, 2021). Although investigations of sexually-dimorphic FASD outcomes mediated by microbiota are currently lacking, biological sex has been shown to influence the gut-brain axis in individuals with other neurodevelopmental disorders. One research group has argued that the relationship between ASD and microbiota is heavily influenced by reduced variability in food preference (Yap et al., 2021). Others have found that the degree of microbiome diversity between individuals with and without ASD is mediated by sex, in humans (Wang et al., 2019) and rodents (Coretti et al., 2017), further supporting the need to investigate the interactions between multiple subject factors in individuals with FASDs. The role of biological sex underlying gut-brain axis function in individuals with other neurodevelopmental and neuropsychiatric disorders, including ADHD, anxiety, and depression, has been interrogated in a separate review article (Shobeiri et al., 2022). Given the comorbidity of FASD and other neurodevelopmental and neuropsychiatric disorders (e.g., Kambeitz et al., 2019; Coles et al., 2022), as well as common physical and behavioral symptoms across these disorders, it is likely that sex contributes to distinct microbiota alterations in individuals with FASDs.

## Author contributions

DU, SR, and AM contributed to conception and design of the review. DU, SR, AB, EL, RK, and AM wrote sections of the manuscript. RM and AM revised the work critically for important intellectual content. All authors contributed to manuscript revision, read, and approved the submitted version.

## Funding

This work was supported by the National Institute on Alcohol Abuse and Alcoholism (F32AA029866, SR) and by Texas A&M University’s Accountability, Climate, Equity, and Scholarship (ACES) Faculty Fellows Program (AM).

## Conflict of interest

The authors declare that the research was conducted in the absence of any commercial or financial relationships that could be construed as a potential conflict of interest.

## Publisher’s note

All claims expressed in this article are solely those of the authors and do not necessarily represent those of their affiliated organizations, or those of the publisher, the editors and the reviewers. Any product that may be evaluated in this article, or claim that may be made by its manufacturer, is not guaranteed or endorsed by the publisher.

## Footnotes

^1^In rodent models, the *in utero* period (gestational days 1–18 for mice and 1–21 for rats) is roughly equivalent to the first and second trimesters of human development while the early postnatal period (post-natal days 1–10) is roughly equivalent to the third trimester of human development (Workman et al., 2013).

^2^For zebrafish development 24 hpf is roughly equivalent to up to 8 gestational weeks in humans and 48 hpf is roughly equivalent to 16 gestational weeks (Walter et al., 2019).
